# Supplementary material for: An Oligocene giant rhino provides insights into Paraceratherium evolution
Source: Commun Biol. 2021 Jun 17;4:639. doi: 10.1038/s42003-021-02170-6 (PMC8211792; doi:10.1038/s42003-021-02170-6)
Supplement: Supplementary file 2 — Supplementary Information [file 42003_2021_2170_MOESM2_ESM.pdf]

Supplementary Information for:

An Oligocene giant rhino provides insights into *Paraceratherium* evolution

Tao Deng, Xiaokang Lu, Shiqi Wang, Lawrence J. Flynn, Danhui Sun, Wen He & Shanqin Chen

Supplementary Note 1

Geologic Setting of the Linxia Basin

The Linxia Basin is located on the triple-junction of the northeastern Tibetan Plateau, western Qinling Mountains and the Loess Plateau, delineated by high angle deep thrusts (Fig. 3). The lateral extent of the Linxia Basin is marked by structural boundaries on the northern, western, and southern edges, but its eastern margin is poorly determined. The basin is filled with 700-2000 m of late Cenozoic deposits, mainly red in color and dominated by lacustrine siltstones and mudstones punctuated by fluvial conglomerates or sandstones, and 30-200 m of Quaternary loess sediments. The Yellow River and its main tributaries, the Daxia and Tao rivers, intermittently incise the whole late Cenozoic strata, which provide good access for study[1]. To the west and south, the major basin-bounding faults within the Tibetan Plateau are the Leijishan and North Qinling faults, respectively. The Cenozoic deposits of the Linxia Basin begin with late Eocene deposits, lapping over Cretaceous deposits in the Maxian Mountain to the north. Throughout the central part of the Linxia Basin, the

oldest deposits were laid down unconformably on granite of presumed Paleozoic age. To the southwest of the Linxia Basin, the Tibetan Plateau consists of Devonian-Permian terrestrial and marine deposits and Triassic submarine fan deposits, which were shed by the east-southeast striking Qinling mountain belt to the east of the plateau[2].

Prior to the 1960s, Cenozoic deposits of the Linxia Basin were simply called the Gansu Series. In 1965, the First Regional Geologic Survey Team of the Gansu Geological Bureau created a new name, Linxia Formation, for the whole Cenozoic sequence, subdivided it into 4 members (1 to 4), and treated the whole sequence as Pliocene based on *Hipparion* fossils found in the upper-most part of the sequence. A new formation, Jiaozigou Formation was created for the lower part of the section[3], corresponding to the members 1 and 2 of the Linxia Formation, thus restricting the Linxia Formation to the members 3 and 4. A research group of Lanzhou University further subdivided the Linxia sequence in the 1990s, nominating a series of new lithological units and dating them mainly by paleomagnetic data[1,4]. The lithological sequence was revised, and the following units were adopted[5-8]: the late Eocene – early Oligocene Tala Formation, late Oligocene Jiaozigou Formation, early Miocene Shangzhuang Formation, early middle Miocene Dongxiang Formation, late middle Miocene Hujialiang Formation, late Miocene Liushu Formation, early Pliocene Hewangjia Formation, late Pliocene Jishi Formation, and the early Pleistocene Wucheng Formation (Fig. 5).

The studied fossils were collected from the Jiaozigou Formation in the lower part

of the Cenozoic strata in the Linxia Basin. Underlying the Jiaozigou Formation, the Tala Formation consists of brownish red sandstones and mudstones, mingled with some penecontemporaneous and secondary gypsum crystals. The Tala Formation is seen only in deep gullies along the eastern bank of the Daxia River in Dongxiang County, such as Maogou, Jiaozigou, and Yagou. The Jiaozigou Formation consists of brownish conglomeratic coarse sandstones with large cross-bedding, yielding the giant rhino fauna in the lower part (Fig. 4) and brownish red silty mudstones with secondary gypsum in the upper part (Fig. 5). The Jiaozigou Formation has the same distribution as the Tala Formation, both exposed in the drainage of the Daxia River, not eastwards across the Nanyang Mountain, the water divide between the Daxia and Guangtong rivers.

Overlying the Jiaozigou Formation, the Shangzhuang Formation consists of yellowish brown carbonate-cemented medium sandstone below, and brownish red silty mudstones above. This formation has a more widespread distribution, expanding to the Guangtong River drainage, but the lower sandstones are not exposed there.

Paleomagnetic dating was done for the Linxia sequence[1,2,4,9], in which the Maogou and Wangjiashan sections cover the Paleogene part, but we disagree with some of those authors' age assignments based on correlations of some mammalian faunas. We reinterpreted the paleomagnetic result[2]. According to our correlation[8], N14 in their Wangjiashan section and N8 in their Maogou section should correspond to C5n.2n. Recent new paleomagnetic measurements support these revisions[9].

The Jiaozigou Fauna (Supplementary Table 1) comes from the sandstones of the

Jiaozigou Formation at Wangjiachuan (Fig. 4), Jiaozigou, Yagou, and Tala in Dongxiang County. The giant rhino is a representative mammal in Asia, and infrequently discovered in Eastern Europe. Giant rhinos diversified in the middle Oligocene, and became much derived in the late Oligocene. Besides the new species *Paraceratherium linxiaense*, the Jiaozigou Fauna also includes other mammalian taxa. The giant rhino *Dzungariotherium orgosense*, first found from the Junggar Basin in Xinjiang, was of large size with rudimentary lower incisors, well-developed antecrochets, and wide foot bones. In Xinjiang, *D. orgosense* coexists with *Lophiomeryx*, and the last record of the latter is from the middle late Stampian age in Europe[3]. *Allacerops*, another rhinocerotid in the Oligocene of Asia, was found from the Oligocene of Lanzhou Basin adjacent to the Linxia Basin[10]. *Schizotherium* was also a characteristic Oligocene form in China, and it was found from the Oligocene Nanpoping Fauna of Lanzhou Basin[11]. *Aprotodon* found only from Pakistan, Kazakhstan, and the Lanzhou Basin, coexisted with the giant rhino in these three regions[9,12] as in the Jiaozigou Formation of the Linxia Basin. *Ronzotherium* was found only in the Oligocene of Eurasia[13]. The entelodont was the most diversified during the Sannoisian and the early-middle Stampian ages, and *Paraentelodon macrognathus* was very abundant in the Jiaozigou Fauna. *Tsaganomys* appeared first in the late Ulantatalian age, and definitive records end in the early Tabenbulukian. As a result, *Tsaganomys* and *Tataromys* are the typical index fossils for the Asian Oligocene[14,15]. *Tsaganomys* and *Tataromys* were found from many Oligocene localities in North China, Mongolia and Kazakhstan[7,14]. Apparently, the age of the Jiaozigou Fauna is

Tabenbulukian, late Oligocene.

As a result, all mammal fossils of the giant rhino fauna from the Linxia Basin show clearly that they belong to the late Oligocene[5,7,8,16,17]. A magnetic age estimate of ~21 Ma (early Miocene) was given for this fauna[2]. Because this fauna can be correlated to the Chinese Tabenbulukian mammal age, its reversed paleomagnetic polarity best matches chron C8r, with an age of 26.5 Ma (Fig. 5).

Supplementary Note 2

Additional Description and Comparison of *Paraceratherium linxiaense*

Skull of MHV 2006 is well-preserved, with M1 much worn close to the crown base and M3 is moderately worn. The right posterior part and orbit are slightly deformed by lateral compression, and the right zygomatic arch and anterior part of nasals are lost (measurements in Supplementary Table 2).

Occipital aspect of skull: The nuchal face is bell-shaped in outline, and narrows in the upper half. The occipital crest is rounded above and gradually inclined laterally, and the upper part of the occipital surface is the widest dorsally, with a width of 125.8 mm. The nuchal ligament depression is a large, deep, inverse triangle with a dorsal width of 86 mm and a height of 128 mm. The nuchal tubercle is very prominent but narrower than the upper border of the foramen magnum. The occipital condyle is higher (125 mm) than wide (90 mm); its lateral margin has a short and steep upper part, and a long and curved lower part, approximately three times the upper part. The upper border of the foramen magnum is semicircular, and lateral borders are vertical and parallel, with a width of 51 mm and a height of 97 mm. The distance of the lateral margins of both condyles is 219.9 mm (Fig. 1e).

Dorsal aspect of skull: There is a shallow sagittal groove along the parietal suture, which is laterally bounded by the prominent parietal crest, and fades out anteriorly at the level of the anterior margin of the temporal condyle where the parietal crest on each side diverges anteriorly and they finally fuse to the lateral edge of the frontal bone. The smallest width between the parietal crest is located anterior to

the nuchal crest, about 34.4 mm. The frontals are constricted at the middle of temporal fossa, and their sagittal part is gradually convex anteriorly while lateral parts are concave. The postorbital process is weak, and the supraorbital tubercle is absent. The widest position of the dorsal surface is located between the supraorbital tubercles. The nasal bone process is narrow, long, with an M-shaped cross section; the transition from its basement to its process is abrupt and steep, giving a wide area anterior to the orbit. The posterior border of the nasal notch has a marked angle from the maxillary surface, at which nasals have a width of 111.55 mm much narrower than that of the nasal notch below it. The posterior part of the premaxillary bone flattens on the inner surface of the maxillary. The premaxillary bone is laterally flattened, and has a nearly vertical profile in anterior view. The right and left premaxillary bones are fused anteriorly and completely, with a thick, rough, and gear-shaped anterior margin, at the lower corner of which, the incisor extends antero-inferiorly at an angle of 45°. The incisor fission is absent (Fig. 1c).

Lateral aspect of skull: The dorsal skull profile has a highly raised occipital part, with the dorsal profile nearly parallel to the zygomatic arch. Due to deformation, it is unknown whether the process of nasal bone has a dorsally arched profile. The occipital crest projects posteriorly, but does not exceed the occipital condyle. The nuchal face inclines forward in an angle of 60°. The occipital crest fades out downward at the lateral face of the strong posttympanic process, and its branch anteriorly continues to the temporal crest above the upper rim of the external auditory meatus. The paraoccipital process and tympanic process comprise a very thick and

strong process, not tapered ventrally, with an upper width of 128 mm and a lower width of 95 mm. The paraoccipital process has a height of 123 mm, and its ventral end is 66 mm below the lower margin of the occipital condyle. The ventral surface of the paraoccipital-tympanic process is anteriorly narrow (12 mm) and posteriorly wide (33mm). The postglenoid process is robust, with vertical upper 1/3 and antero-inferiorly inclined lower 2/3, and the antero-posterior width is 83 mm at their transition. There is a large space between the postglenoid and the tympanic-paraoccipital processes, 45 mm long at their lower ends. The zygomatic arch below the orbit is horizontal and robust, then slender and upturned, with a rough lateral surface. The postorbital process is weak on the zygomatic process. The anterior margin of the orbit is situated above the middle of M2, placing the alveolar margin horizontally. The lachrymal tubercle is located superiorly in the front of the orbit. The infraorbital foramen is rounded and situated above the P4/M1 boundary, facing antero-laterally, with a height of 36 mm, a length of 45 mm, and a horizontal distance of 113.5 from the posterior margin of the nasal notch. The posterior half of the lateral margin of the nasals droops gradually. Under the nasal notch, a 34 mm part of the maxillary bone is exposed.

The cross section of the nasals is flatly lenticular, with the maximum thickness at the sagittal suture. There is a rounded spongy area in the central part of each nasal. The maxillary surface is smooth, and its upper margin (lower margin of the nasal notch) has a narrow and sharp posterior part. The nasal notch has an asymmetrical V-shaped outline in lateral view, with its upper border nearly straight. The posterior

border of the nasal notch has a distance of 126 (left) and 132.5 (right) mm from the infraorbital foramen or 296 mm from the alveolar margin respectively. The premaxillary from front of the P3 turns above the maxillary gradually and becomes rough, thick, and convex, with a height of 84.5 mm behind I1. The maxillary-premaxillary suture is inclined antero-inferiorly and ends at 109 mm in front of P2. The muzzle is uplifted in lateral view, with a rounded gear-like anterior end. There is a sagittal gap at the dorsal aspect of the fused part of the left and right premaxillary bones. The anterior rough area above I1 has a distance of top 46 mm and lower 38 mm between premaxillary bones, and a height of 74 mm. The deep valley between premaxillary bones has an anterior with of 35 mm and a posterior width of 10 mm. The diastema on the maxillae from the incisor to P2 is 139 mm. The alveolar margin between two I1 is 24 mm wide. The muzzle is 94 mm (right) high in front of P2. The alveolar margin around I1 is bulges so that the alveolus is 26 mm wide and 32 mm long antero-posteriorly (Fig. 1a).

Ventral aspect of skull: The maximum width of the choanae is seriously compressed. The posterior margin of the pterygoid extends backward and dorsally at an obtuse angle. The anterior end of the temporal fossa slightly passes the posterior border of M3. The temporal condyle is slightly convex in lateral view and slightly concave forward in ventral view. The facet behind the glenoid fossa is triangular in shape, with a projecting edge. The postglenoid process is robust and antero-posteriorly compressed. The basilar tubercle is wide, low, rough, and has a sagittal crest. The posterior region of the basioccipital is wide, flat, and slightly

convex.

The posttympanic process is well developed and fused with the postglenoid process to become antero-posteriorly compressed. The external auditory meatus is unclosed and the temporal condyle is straight and flat. The flat palate has a posterior margin at the level of the middle of M3. The maxillary tubercle is large (Fig. 1b).

Upper teeth: I1 is strongly rounded and cone-shaped, projecting antero-inferiorly at an angle of 45°. Its root is robust and exposed. The cross section has a diameter of 26 mm long and 20.5 mm wide. The surface of I1 has vertical enamel lines, strong in posterior aspect and absent in lateral aspect. DP1 is absent (Fig. 1b; measurements in Supplementary Table 3).

P2 is triangular in occlusal surface, with an undulated labial wall. The parastyle fold is wide and shallow, and the paracone rib is weak. The protoloph is narrow and slightly widened from labial to lingual, and curved posteriorly. The lingual margin of the protocone is rounded. The metaloph is slender in middle and intersects the anterior end of the hypocone. The hypocone is rounded, expanded, and much larger the protocone. The median valley is closed to become a large and wide triangle. The lingual valley is deeply V-shaped. The posterior valley is broad. The anterior and lingual cingula are continuous and strong, and the labial cingulum is complete.

P3: The labial wall is shallowly undulated, with weak parastyle fold and paracone rib. The protoloph is wide, expanded lingually, and inclined posteriorly to close the triangular median valley that is wider than long. The metaloph is slender, lingually expanded, and narrow at the connection with the anterior corner of the

hypocone. The lingual valley is wide and shallow. The lingual cingulum is complete and surrounds the lingual half of the tooth. The labial cingulum is well developed. The posterior valley is wide, with a convex bottom.

P4: The labial wall is shallow but more undulated than that of P3. The lingual margin is rounded. The metaloph is slightly wider than that of P3, and its lingual part is curved anteriorly and connects the anterior corner of the hypocone. The closed median valley is a transversely wide triangle. The crista is weakly convex. The lingual valley is wide and shallow. The anterior, lingual, and posterior cingula are continuous and thick. The labial cingulum is strong and undulated.

M1: The left M1 is much worn, and the right M1 is lost. The labial wall is straight and inclined postero-lingually. The protocone is expanded, with a rounded lingual margin. The lingual valley is wide and deep. The labial cingulum is complete and strong.

M2: The labial wall is slightly undulated and strongly inclined postero-lingually, with the marked parastyle fold and strong parastyle rib. The protoloph is wide and inclined posteriorly, with a large and triangular antecrochet that reaches the metaloph and almost closes the median valley. The protocone is expanded, with rounded lingual margin. The anterior valley is wide, deep, and V-shaped. The metaloph is wide and constricted at the connection with the ectoloph. The hypocone is moderately large and rounded, with an anterior constriction. The lingual cingulum surrounds the protocone. The labial cingulum is strong and complete.

M3: The outline is triangular in occlusal surface. The large and rounded

parastyle is marked, and the parastyle rib is very strong. The ectometaloph is wide and curved. The protoloph is wide and slightly inclined. The antecrochet is convex. The protocone is moderately large. The median valley is open and broad. The hypocone is larger than the protocone. The anterior, lingual, and posterior cingula are continuous and thick, but low at the entrance of the median valley. The labial cingulum is strong and undulated.

Mandible: It is relatively complete, only lacking the mandibular angle, mandibular symphysis, and premolars of the right ramus. The horizontal ramus is low in height and long. The lower margin is shallowly concave and nearly straight under the cheek teeth, with the uplifted mandibular symphysis and deep vasal notch anterior to the mandible angle. The upper margin of the mandibular symphysis is straight at the diastema whose length is longer than 135 mm. The posterior border of the mandibular symphysis is situated at the level of the p4/m1 boundary. The oval mental foramen is small with antero-posterior diameter of 24 mm, and located in the lower half of the horizontal ramus under the paraconid of p4. The ascending ramus is relatively high with a height of 547 mm at the coronoid process, 488 mm at the condyloid process. The mandibular condyle is transversely extended with a width of 189 mm, corresponding to the length of the glenoid fossa of the skull. The medial end of the condyloid process is curved posteriorly. The lateral half of the condyle is slightly inclined anteriorly. The notch between the coronoid and condyloid processes is wide and deep. The lower part of the coronoid process is wide antero-posteriorly, and the upper part above the condyloid process tapers gradually as it curves

posteriorly, with rounded anterior margin and angularly concave posterior margin. The posterior margin of the ascending ramus is slightly curved dorsally and anteriorly inclined, and in posterior view, it is wide and rough, with a medially curved upper apophysis. The mandibular angle is rounded, with many weak radial ridges. On the lateral surface of the ascending ramus, the masseter fossa is very deep under the coronoid process and occupies most of the surface so the surrounding margins are highly uplifted. The medial surface of the ascending ramus is shallowly depressed. The mandibular foramen is very large and situated anteriorly, its bottom at the alveolar level. The groove behind the mandibular foramen is deep, long, and extending upward (Figs. 1f-h; measurements in Supplementary Table 4).

Lower teeth: The p2 is lost, and its alveolus is single-rooted with a length of 27 mm and a width of 24 mm. Due to damage of the anterior part of mandible, incisor is not accessible, but its crushed medial face at the anterior part exposed a section of the alveoli of i2 in squint angle view, whose location is at the lower part of symphysis and has an antero-posteriorly elongated rounded outline, with a height of 24 mm (Fig. 1g; measurements in Supplementary Table 5).

p3: the occlusal surface is trapezoid, with slightly shorter anterior margin than the posterior one, and double-rooted. The postero-labial corner of the protoconid is rounded. The labial groove is shallow. The labial cingulum is well developed and V-shaped, with the bottom behind the labial valley and uplifted anterior and posterior branches whose ends reach the occlusal surface respectively. The lingual cingulum is complete. The paralophid is a small triangle. The metalophid is robust, much wider

than the endolophid. The anterior valley is shallowly V-shaped, and the posterior valley is deeply U-shaped and anteriorly inclined.

p4: The occlusal surface is nearly rectangular. The postero-labial corner of the protoconid is more angular than that of p3. The labial groove is wide and deep. The labial cingulum is strong but absent under the protoconid. The lingual cingulum is complete. The paralophid is short and wide. The metalophid is robust, wider and shorter than the endolophid. The anterior valley is narrowly V-shaped and posteriorly inclined, while the posterior valley is deeply U-shaped and anteriorly inclined.

m1: It is much worn. The occlusal surface is trapezoid with slightly shorter lingual side than the labial side. The postero-labial corner of the protoconid is nearly right-angled. The labial groove is wide and deep. The labial cingulum has some pillars and a plate at the labial valley, but it is absent under the protolophid. A rudimentary lingual cingulum is present on entrances of anterior and posterior valleys.

m2: The occlusal surface is rectangular. The postero-labial corner of the protoconid is right-angled. The labial groove is deep. The labial cingulum is strong and slightly W-shaped, but absent under protoconid. The lingual cingulum is the same as m1. The paralophid is triangular. The metalophid is robust but narrower than the endolophid. The anterior valley is widely V-shaped and the posterior valley is deeply U-shaped. Both the protolophid and hypolophid are slightly lingually oblique.

m3: On the labial wall, the vertical lines are marked. The occlusal surface is trapezoid, with slightly shorter anterior margin than the posterior one. The postero-labial corner of the protoconid is rounded. The labial groove is very deep and

wide. The labial cingulum is strong, but absent under the protoconid and near the root under the hypolophid. The lingual cingulum is the same as m1-2. The posterior cingulum is continuous with the labial and lingual ones, and it is very strong to form a prominent brim. The paralophid is triangular. The metalophid is robust, slightly wider than the endolophid. The anterior valley is shallowly V-shaped, and the posterior valley is deeply U-shaped, straight anteriorly, with curved postero-lingual margin.

Atlas: The vertebral foramen is nearly quadrate in anterior view, but mushroom-shaped in posterior view. The anterior articular cavity for the occipital condyle is protruded anteriorly, like a short gun barrel. The wing is rather elongated backward and laterally. Two transverse foramens flank the lateral-superior corner of the posterior articular process facet. The posterior articular facet is flat or slightly concave (Fig. 2a; measurements in Supplementary Table 6).

The much larger size of the atlas of *P. linxiaense* than *P. bugtiense* and *P. lepidum* results from the further posteriorly and laterally extended wing, making the widest position of the atlas backward to between the posterior end of the wing, rather than its lateral edge in the latter two species. The anteriorly protruded part of the atlas containing the anterior articular cavities is at the anterior 1/4 of the atlas in *P. linxiaense*, but much shorter than those of *P. bugtiense*, *P. asiaticum*, and *P. lepidum*. This is resulted from the different outline and size of the atlas of *P. linxiaense*, indicated a stronger skull-atlas joint.

Axis: it has an outline much longer than wide. The odontoid process is short and strong. The vertebral fossa is dumb-bell shaped, with both the dorsal and ventral rims

slightly concave. The most posterior end of the axis is at the ventral rim of the vertebral fossa. The spinous process is low; its anterior end is flush with the prezygapophysis, and the posterior end is not reaching the dorsal edge of the vertebral fossa. The intervertebral foramen is a large and laterally flattened fissure. The transverse process extends backward to the level of the dorsal rim of the vertebral fossa, between which is the widest part of the axis (Fig. 2b; measurements in Supplementary Table 6).

The axis of *P. linxiaense* shares many features with *P. grangeri* and *P. lepidum*, which characterize *Paraceratherium*, such as the dumb-bell shaped vertebral fossa. *P. linxiaense* resembles *P. grangeri* in many aspects: the transverse process has a posterior end before the vertebral fossa; in lateral view, the lowest point of the axis is at the ventral edge of the vertebral fossa. On the other hand, similar to *P. lepidum*, the axis of *P. linxiaense* has a spinous process with both the anterior and posterior ends at the same horizontal level, which is derived relative to the highly raised posterior end of the spinous process in *P. grangeri*. Meanwhile, the new axis is more derived than *P. lepidum* because of the nearly straight dorsal profile of the spinous process, which is obviously arched in the latter.

Thoracic vertebrae: The caput is much wider than height. The vertebral fossa is triangular in outline, with a straight dorsal edge, and much wider than high. On the transverse process, the facet for the tubercle of the rib is nearly circular, deeply concave. The anterior costal facet and the posterior one are large, deeply concave, and isolated to the caput and the fossa, respectively (Fig. 2c; measurements in

Supplementary Table 6).

The articulated thoracic vertebrae of *P. linxiaense*, like *P. grangeri*, *P. asiaticum*, and *P. lepidum*, have a large body, triangular vertebral fossa, high spinous process, large facets for the rib. In *P. linxiaense*, no incision is present on the dorsal edge of either the caput or the vertebral fossa of the thoracic vertebrae, and its body length is shorter than those of *P. grangeri*, *P. asiaticum*, and *P. lepidum*.

Supplementary Note 3

Paleogeographical literature survey

The Tarim Basin between the India-Asia collision zone and the Tianshan accretional orogeny has a close relationship with the uplift and the dynamics of Tibetan Plateau. During the Mesozoic and Cenozoic time, it underwent a growth of the foreland basin, including Kuqa, Awat, Southwestern Tarim, and Southeastern Tarim foreland basins, surrounded by a paleo-archipelago environment of the Paleo-Asia Ocean and Tethys Ocean[18,19]. Many researchers have confirmed the extensive presence of the marine and lacustrine environment in these basins[20-24].

The Cenozoic sediments occur in three regions, including the southwestern, southeastern, and northern Tarim. Stratigraphy of the first region has been extensively investigated and divided into the Kashi Group, the Wuqia Group, and the Artux Formation in ascending order[22,25]. The Paleogene strata of the western Tarim are the Kashi Group, including the Aertashi, Qimugen, Kalatar, Wulagen, and Bashibulake formations, and the overlying Neogene Wuqia Group includes the Keziluoy, Anjuan, Pakabulake formations[26]. Meanwhile, due to discovery of the foraminifer fossils with special features, the lower part of the former Oligocene Bashibulake Formation was designated as the Zhuoyolegansu Formation, and considered latest Eocene in age[22,27]. The contact between the Oligocene Bashibulake Formation and the early Miocene Keziluoyi Formation is disconformity, and the onset of the Keziluoyi Formation at the northern margin of the western Tarim remains uncertain[21,24].

It currently recognizes three regression records in the Paleogene of the

southwestern Tarim Basin, including top of the Qimugen Formation, top of the Ulagen Formation, and the forth-fifth members of the Bashibulake Formation[22,24,28-31].

It is widely noted that, after the fifth regression in the late Oligocene, the marine or lacustrine depositions in the Tarim Basin have persisted to the Miocene as indicated by the fossil records and the stable isotope data[20,21]. The Miocene foraminifer assemblages from the Keziluoyi Formation in Wuqia, the Anjuan Formation in Wuqia and Yingjisha, the Jidike and Kangcun formations in Luntai indicate that the marine or at least brackish water environment was present in western and northern Tarim Basin during the early-middle Miocene[32,33]. The foraminifer assemblage from the Miocene strata along the Miran River in the southeastern Tarim Basin, whose age was confirmed by data of the apatite fission track and (H-Th)/He ages and the thermal models, contains the planktonic taxa, indicating a depositional environment connecting to the open marine water[30].

Most areas of the Tarim Basin were dominated by the fan-deltas of the early phase and littoral to the shallow lacustrine environment at the transgression intervals and the early Neogene when the Tarim Basin underwent rapid subsidence[20-23]. Most area of the Tarim Basin was covered by the marine or the lake water during the Paleogene until the Neogene period, although the Miocene transgression remains denied by some authors.

Furthermore, a new research finds that the marine feature of the southern Tarim is stronger than the northern based on the different foraminifers in the Late Cretaceous strata of two regions, with wider distribution scope of the marine strata

than conventionally recognized, and implies that the transgression could be from the West Kunlun Sea, invading from south to north in this basin[34]. These results indicate complex marine incursion in the southeastern Tarim, probably extending to the eastern Tarim if the incursion of the sea water was along the West Kunlun, extending northward and eastward.

On all accounts, regardless of the scope of the marine transgression and the lacustrine environment in the Tarim Basin, it is impossible for the land mammals to migrate between its north and south coasts (Fig. 7; Supplementary Fig. 3).

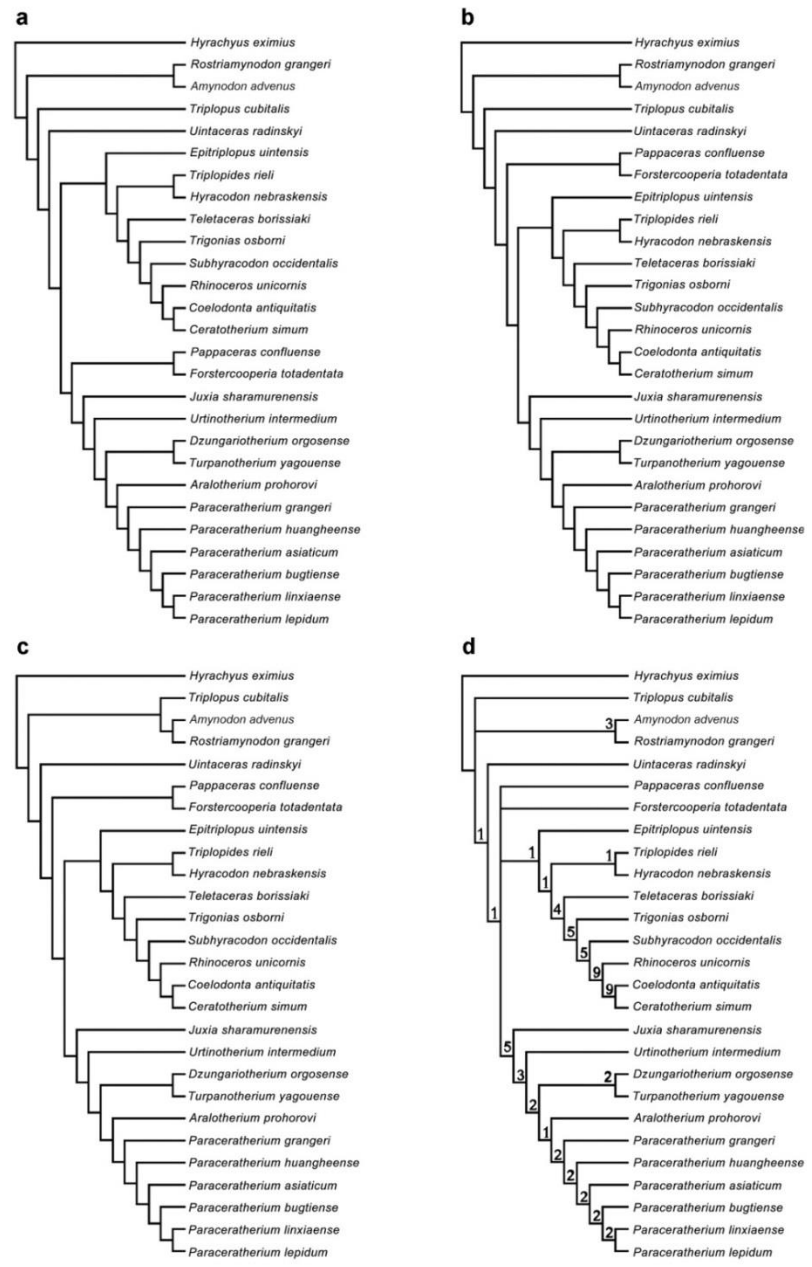

Supplementary Figure 1. Parsimonious trees of partial analysis based only on cranial and teeth characters of 27 taxa, using TNT 1.5 (tradition search with the strategy of WAG and TBR, 100 random seed, 1000 replications, length 233, CI 0.54, RI 0.79). **a**, **b**, **c**, are three best trees, and **d** is their consensus tree with the Bremer support numbers nearby the node.

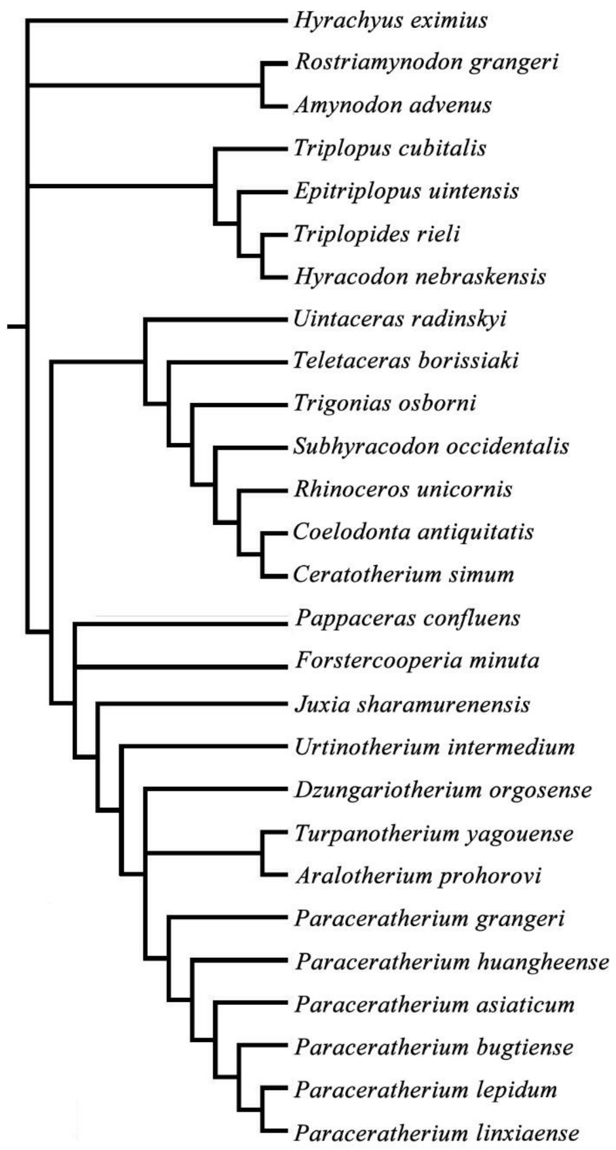

Supplementary Figure 2. Likelihood estimation of relationship of the giant rhino, based on the same matrix (27 taxa, 155 character) and using Mrbayes 3.2.7 (lset nst=6, rates=invgamma, ngen=10000000). Within clade of *Paraceratherium*, six species display a relationship similar to the result of the parsimonious analysis, but the relationships among four late Oligocene genera of the giant rhino are not resolved. The relationship of the Paleogene rhinocerotoids is also ambiguous in the likelihood estimation.

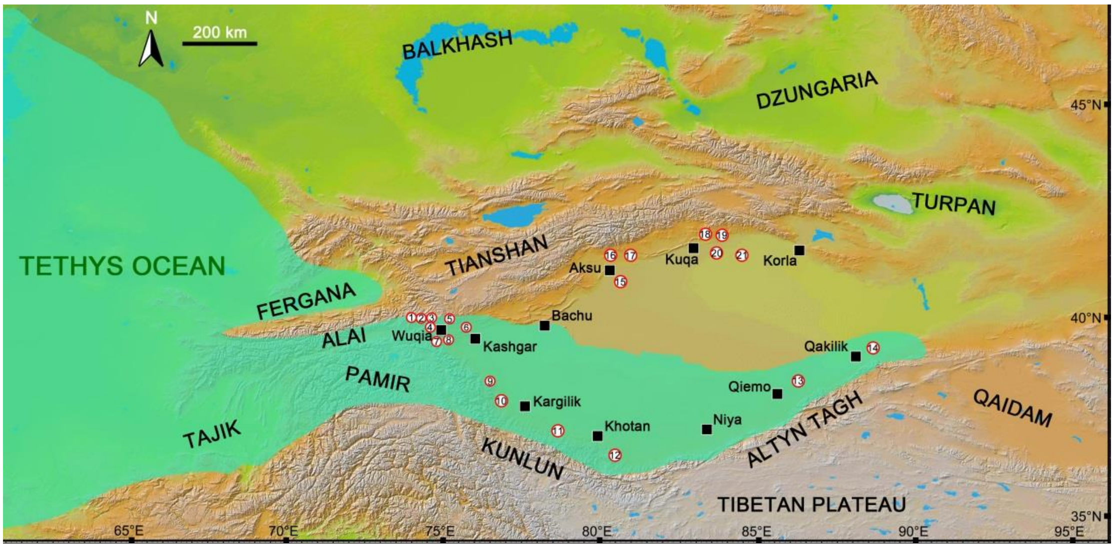

Supplementary Figure 3. Transgression of the Tethys Ocean into the Tarim Basin in the Oligocene, modified from a research[35] and Deep Time Maps (https://deeptimemaps.com), and numerous researches of the Tarim Basin. The classic Cenozoic sections in the Tarim Basin are marked using numerical symbols: 1, Urukechati; 2, Bashibulake; 3, Kangsu; 4, Wulagen; 5, Kuzigongsu; 6, Xiaoatushi; 7, Pakabulake; 8, Keziluoyi; 9, Qimugen; 10, Aertashi; 11, Keliyang; 12, Arqik; 13, Janggalsay; 14, Miran River; 15, Awat; 16, Talake; 17, Xiaokuzibai; 18, Yiqikelike; 19, Tugerming; 20, Jianrengou; 21, Ayikumuqi.

Supplementary Table 1. List of Mammalian taxa of the late Oligocene giant rhino fauna from the Jiaozigou Formation of the Linxia Basin in Gansu Province, China

Rodentia
Tsaganomyidae
Tsaganomys altaicus
Ctenodactylidae
Tataromys sp.
Creodonta
Hyaenodontidae
Megalopterodon sp.
Perissodactyla
Chalicotheriidae
Schizotherium ordosium
Hyracodontidae
Hyracodontidae gen. et sp. indet.
Ardynia altidentata
Ardynia sp.
Allacerops sp.
Paraceratheriidae
Dzungariotherium orgosense
Turpanotherium yagouense
Paraceratherium linxiaense sp. nov.

Rhinocerotidae
Ronzotherium sp.
Aprotodon lanzhouensis
Artiodactyla
Entenodontidae
Paraentelodon macrognathus

Supplementary Table 2. Cranial measurements[36] (in mm) of *Paraceratherium linxiaense* and comparisons with other giant rhinos.

measures	*P. lin*	*P. lep*	*P. gra*	*D. org*
1 Distance between occipital condyle and premaxilla	1158	1325*	1286	1210
2 Distance between occipital condyle and nasal tip	>936	>1033[#]	-	1073
3 Distance between nasal tip and occipital crest	>831	>998	-	1015
4 Distance between nasal tip and bottom of nasal notch	>208	>179[#]	380[#]	237
5 Minimal width of braincase	-	-	-	-
6 Distance between occipital crest and postorbital process	528*	533[#]	610[#]	-
7 Distance between occipital crest and supraorbital process	593*	597[#]	-	-
8 Distance between occipital crest and lacrimal tubercle	638*	669[#]	-	-
9 Distance between nasal notch and orbit	39	52[#]	-	-
13 Distance between occipital condyle and M3	520	622[#]	-	-
14 Distance between nasal tip and orbit	>271	>224[#]	-	216
15 Width of occipital crest	>138	227[#]	-	-
16 Width between mastoid processes	232	293[#]	-	-
17 Minimal width between parietal crests	34	106[#]	-	-
18 Width between postorbital processes	260*	277[#]	-	-
19 Width between supraorbital processes	331*	410[#]	-	-
20 Width between lacrimal tubercles	312*	367[#]	-	-
21 Maximal width between zygomatic arches	>446	>460	614	543*
22 Width of nasal base	114	127[#]		-

23 Height of occipital surface	210*	243#	216	-
25 Cranial height in front of P2	331*	361#	-	-
26 Cranial height in front of M1	327*	384#	-	-
27 Cranial height in front of M3	390*	394#	-	-
28 Width of palate in front of P2	121*	-	-	-
29 Width of palate in front of M1	117*	-	-	-
30 Width of palate in front of M3	83*	-	-	-
31 Width of foramen magnum	68	80#	-	-
32 Width between exterior edges of occipital condyle	219	270#	306	-

Measures: *Paraceratherium lepidum*[17], *P. grangeri*[37], and *Dzungariotherium orgosense*[38]. * means the measures are based on damage part of specimens, and # means measures are based on figures.

Supplementary Table 3. Measures (in mm) of upper cheek teeth (length/width) and incisor (height/length) of *Paraceratherium linxiaense* and comparisons with other giant rhinos.

Measures	*P. lin*		*P. bug*	*P. lep*		*P. asi*	*P. hua*	*P. gra*	*D. org*	*A. pro*	*U. int*	
	L	R		L	R						L	R
I1	33/23	32/19	-/-	-/-	-/-	-/-	-/-	>65/70[#]	-/20	-/-	-/-	-/-
P2	52/64	52/64	44/-	45/59	50/60	36/50	56/63	45/58	57/67	-/-	-/-	-/-
P3	56/87	59/86	53/64	60/86	67/88	35/70	70/87	58/83	58/89	-/-	45/68	45/67
P4	70/96	73/96	59/66	68/93	70/96	50/75	80/105	60/94	65/107	-/-	53/77	53/77
M1	69/92	-/-	68/73	-/101	73/103	75/-	98/124	77/98	86/109	-/-	56/88	51/92
M2	93/98	90/91	-/-	98/112	106/112	80/75	88/113	95/103	90/117	96-108/92-111	73/102	73/105
M3	87/98	89/99	-/-	96/104	-/110	-/80		88/100	99/110	78-90/97-107	-/-	-/-

Measures: *Paraceratherium lepidum*[17], *P. bugtiense* from skull A[39], *P. asiaticum*[40], *P. grangeri*[37], *Dzungariotherium orgoense*[38], and *Aralotherium prohorovi*[41]. [#] means measures are based on figures.

Supplementary Table 4. Mandibular measures[36] (in mm) of *Paraceratherium linxiaense* and comparisons with other giant rhinos.

Measures	*P. lin*	*P. lep*	*P. bug*	*P. asi*	*P. hua*	*P. gra*	*U. int*
1 Length	820*	855	720	630	-	830#	715
2 Distance between posterior borders of symphysis and ascending ramus	590*	615#	656	-	-	-	675
3 Height of horizontal ramus in front of p3	122	-	-	-	-	-	-
4 Height of horizontal ramus in front of p4	107	-	-	-	-	-	-
5 Height of horizontal ramus in front of m1	99	113#	123	-	-	-	113
6 Height of horizontal ramus in front of m2	101	124#	-	-	-	-	-
7 Height of horizontal ramus in front of m3	121	150#	-	-	-	-	-
8 Height of horizontal ramus behind m3	184	181#	-	-	-	-	-
9 Distance between horizontal ramus in front of m1	97	-	-	-	139	-	-
10 Distance between horizontal ramus in front of m3	105	-	-	-	-	-	-
11 Length of symphysis	229*	219#	212	-	230	-	146
13 Antero-posterior diameter of ascending ramus	209	196#	-	-	-	-	-
14 Transverse diameter of condyle	179	205#	-	-	-	-	-
15 Height at condyle	487	425	-	>340	-	400#	295
16 Height at coronoid process	548	531#	-	-	-	453#	354

Measures: *Paraceratherium lepidum*, *Paraceratherium bugtiense*, *Paraceratherium grangeri*, and *Urtinotherium intermedium*[17]; *Paraceratherium asiaticum*[40]; and *Paraceratherium huangheense*[42]. * means the measures are based on damage part of specimens, and # means measures are based on figures.

Supplementary Table 5. Measures (in mm) of lower cheek teeth (length/width) and incisor (height/length) of *Paraceratherium linxiaense* and comparisons with other giant rhinos.

Measures	*P. lin*		*P. lep*	*P. bug*	*P. asi*	*P. hua*	*P. gra*	*D. org*	*A. pro*	*U. int*
	L	R								
i1	-/-	-/-	-/-	59/55	-/-	39/25	45/39	-/-	37-55/32-41	44/37
p2	-/-	-/-	35/23	29/18	32/20	35/22	34/24	-/-	29-33/21-24	32/24
p3	54/43		52/42	48/37	41/27	48/30	51/47	-/-	43-46/37-39	43/35
p4	66/50	49/27	65/54	57/45	46/30	62/46	57/42	74/57	55-63/44-50	48/40
m1	69/49	66/50	73/53	58/45	63/40	79/57	77/55	87/71	63-73/48-56	60/45
m2	85/56	85/60	94/62	70/45	70/43	96/66	92/60	99/67	71-83/50-60	66/48
m3	91/55	90/57	110/63	79/49	70/45	93/60	89/55	101/65	72-96/50-60	66/53

Measures: *Paraceratherium lepidum* and *Aralotherium prohorovi*[17]; *P. bugtiense*[43]; *P. asiaticum*[40]; *P. huangheense*[42]; *P. grangeri*[37]; *Dzungariotherium orgoense*[38]; and *Urtinotherium intermedium*[44].

Supplementary Table 6. Measures (in mm) of vertebrae of *Paraceratherium linxiaense*.

Measures	1th C. v.	2th C. v.	4th T. v.	5th T. v.
Maximum length	473	>585	141	121
Maximum width	657	419	>366	>340
Maximum height	325	233	-	-
Length of anterior costal facet	-	-	66	-
Width of anterior costal facet	-	-	61.5	-
Width of anterior articular facet	151	144	96	-
Length of anterior articular facet	-	-	126	-
Height of anterior articular facet	138	104	-	-
Width of head	-	-	156	-
Height of head	-	-	106	-
Width of vertebral foramen	147	157	45*	42*
Height of vertebral foramen	135	100	59*	58*
Width of transverse process	657	420	-	-
Length of transverse process	313	-	-	-
Length of rib tubercle facet	-	-	78*	88
Width of rib tubercle facet	-	-	65	65
Width of spinous process	-	138	100	>86
Height of spinous process	-	-	585	566
Length of spinous process	252	-	176	174

Width of cavity	-	284	-	191
Height of cavity	-	-	-	141
Length of posterior costal facet	-	-	77	70
Width of posterior costal facet	-	-	62	52
Width of posterior articular facet	-	-	62	52
Length of posterior articular facet	171	-	77	64
Height of posterior articular facet	116	-	-	-
Length of dorsal arch	252	-	-	-
Length of ventral body	170	-	-	-

* Measures based on damaged or deformed specimens. Abbreviations: C. v., cervical vertebrae; T. v., thoracic vertebrae.

Supplementary References

1. Li, J.-J. et al. *Uplift of Qinghai-Xizang (Tibet) Plateau and Global Change* (Lanzhou Univ. Press, Lanzhou, 1995).
2. Fang, X.-M., Garzione, C., Van der Voo, R., Li, J.-J. & Fan, M.-J. Flexural subsidence by 29 Ma on the NE edge of Tibet from the magnetostratigraphy of Linxia Basin, China. *Earth Planet. Sci. Lett*. **210**, 545–560 (2003).
3. Qiu, Z.-X., Xie, J.-Y., & Yan, D.-F. Discovery of some early Miocene mammalian fossils from Dongxiang, Gansu. *Vert. PalAsiat.* **28**, 9–24 (1990).
4. Fang, X.-M., Li, J.-J., Zhu, J.-J., Chen, H.-L. & Cao, J.-X. Division and age dating of the Cenozoic strata of the Linxia Basin in Gansu, China. *Chin. Sci. Bull.* **42**, 1457–1471 (1997).
5. Deng, T., Wang, X.-M., Ni, X.-J., Liu, L.-P. & Liang, Z. Cenozoic stratigraphic sequence of the Linxia Basin in Gansu, China and its evidence from mammal fossils. *Vert. PalAsiat.* **42**, 45–66 (2004).
6. Deng, T., Wang, X.-M., Ni, X.-J. & Liu, L.-P. Sequence of the Cenozoic mammalian faunas of the Linxia Basin in Gansu, China. *Acta Geol. Sin*. **78**, 8–14 (2004).
7. Qiu, Z.-X., Wang, B.-Y. & Deng, T. Mammal fossils from Yagou, Linxia Basin, Gansu, and related stratigraphic problems. *Vert. PalAsiat.* **42**, 276–296 (2004).
8. Deng, T., Qiu, Z.-X., Wang, B.-Y., Wang, X.-M. & Hou, S.-K. in *Fossil Mammals of Asia: Neogene Biostratigraphy and Chronology* (eds. Wang, X.-M., Flynn, L. J. & Fortelius, M.) 243–273 (Columbia Univ. Press, New York, 2013).

9. Fang, X.-M. et al. Tectonosedimentary evolution model of an intracontinental flexural (foreland) basin for paleoclimatic research. *Global Planet. Change* **145**, 78–97 (2016).

10. Qiu, Z.-X. & Wang, B.-Y., *Allacerops* (Rhinocerotoidea, Perissodactyla), its discovery in China and its systematic position. *Vert. PalAsiat.* **37**, 48–61 (1999).

11. Qiu, Z.-X., Wang, B.-Y. & Xie, J.-Y. Mid-Tertiary chalicothere (Perissodactyla) fossils from Lanzhou, Gansu, China. *Vert. PalAsiat.* **36**, 297–318 (1998).

12. Qiu, Z.-X. & Xie, J.-Y. A new species of *Aprotodon* (Perissodactyla, Rhinocerotidae) from Lanzhou Basin, Gansu, China. *Vert. PalAsiat.* **35**, 250–267 (1997).

13. Heissig, K. Die Rhinocerotidae aus der oberoligozanen Spaltenfullung von Gaimersheim bei Ingolstadt in Bayern. *Abh. Bayer. Akad. Wiss. Math. Nat. Kl.* **138**, 1–133 (1969).

14. Wang, B.-Y. The mid-Tertiary Ctenodactylidae (Rodentia, Mammalia) of Eastern and Central Asia. *Bull. Am. Mus. Nat. Hist.* **234**, 1–88 (1997).

15. Wang, B.-Y. Eocene ctenodactyloids (Rodentia, Mammalia) from Nei Mongol, China. *Vert. PalAsiat.* **39**, 98–114 (2001).

16. Qiu, Z.-X., Wang, B.-Y. & Deng, T. Indricotheres (Perissodactyla, Mammalia) from Oligocene in Linxia Basin, Gansu, China. *Vert. PalAsiat.* **42**, 177–192 (2004).

17. Qiu, Z.-X. & Wang, B.-Y. Paracerathere fossils of China. *Palaeont. Sin.* **29**, 1–396 (2007).

18. Tian, Z.-J. & Song, J.-G. Tertiary structure characteristics and evolution Kuche foreland basin. *Acta Petrol. Sin.* **20**, 7–13 (1999).

19. Xu, Z.-Q. et al. Paleo-Asian and Tethyan tectonic systems with docking the Tarim block. *Acta Petrol. Sin.* **27**, 1–22 (2011).

20. Guo, X.-P. et al. New progress in the study of the marine transgressional events and marine strata of the Meso-Cenozoic in the Tarim Basin. *Acta Geol. Sin.* **76**, 299–307 (2002).

21. Hao, Y.-C. et al. *The Boundary Between the Marine Cretaceous and Tertiary in the Southwest Tarim Basin* (Geol. Publ. House, Beijing, 2001).

22. Jia, C.-Z., Zhang, S.-B. & Wu, S.-Z. *Stratigraphy of the Tarim Basin and Adjacent Areas* (Sci. Press, Beijing, 2004).

23. Shao, L.-Y. et al. Lithofacies paleogeography of the Paleogene in Tarim Basin. *J. Paleoceanogr.* **8**, 353–364 (2006).

24. Wei, H.-H., Meng, Q.-R., Ding, L. & Li, Z.-Y. Tertiary evolution of the western Tarim Basin, Northwest China: A tectono-sedimentary response to northward indentation of the Pamir salient. *Tectonics* **32**, 558–575 (2013).

25. Ding, X.-Z. et al. A study of the Cretaceous-Tertiary sequence stratigraphy of Tarim Basin, Xinjiang. *Acta Geol. Sin.* **23**, 243–248 (2002).

26. Zhou, Z.-Y. & Chen, P.-J. *Stratigraphy in Tarim Basin* (Sci. Press, Beijing, 1990).

27. Compilation Group of Regional Stratigraphic Table in Xinjiang Uygur Autonomous Region. *Northwest Regional Stratigraphic Table: the Xinjiang*

Uygur Autonomous Region Volume (Geol. Publ. House, Beijing, 1981).

28. Yong, T.-S. & Shan, J.-B. The development and formation in the Tarim Bay in Cretaceous-Paleogene ages. *Acta Sedimentol. Sin.* **4**, 67–75 (1986).
29. Tang, T.-F. et al. *Marine Late Cretaceous and Early Tertiary Stratigraphy and Petroleum Geology in Western Tarim Basin*, *China* (Sci. Press, Beijing, 1989).
30. Ritts, B. D. et al. From sea level to high elevation in 15 million years: Uplift history of the northern Tibetan Plateau margin in the Altun Shan. *Am. J. Sci.* **308**, 657–678 (2008).
31. Bosboom, R. E. et al. Timing, cause and impact of the late Eocene stepwise sea retreat from the Tarim Basin (West China). *Palaeogeogr. Palaeoclimatol. Palaeoecol.* **403**, 101–118 (2014).
32. Hu, L.-Y. Late Tertiary foraminifer ecology of the Tarim Basin and its geological significance. *Chin. Sci. Bull.* **27**, 938–941 (1982).
33. Huang, B.-C. et al. Magnetostratigraphic study of the Kuche depression, Tarim Basin, and Cenozoic uplift of the Tian Shan range, western China. *Earth Planet. Sci. Lett.* **251**, 346–364 (2006).
34. Guo, X.-P., Ding, X.-Z., Zhao, Z.-R. & Li, J.-F. The north-south differentiation on the marine feature in the southwest Tarim Basin during middle-late of the Late Cretaceous. *Geol. Rev.* **64**, 1078–1086 (2018).
35. Popov, S.V. et al. 2004. Lithological-paleogeographic maps of Paratethys: 10 maps late Eocene to Pliocene. *Cour. Forsch.-Inst. Senckenberg* **250**, 1-46 (2004).
36. Guérin, C. Les rhinocéros (Mammalia, Perissodactyla) du Miocène terminal au

Pléistocène supérieur en Europe occidentale: comparaison avec les espèces actuelles. *Doc*. *Lab*. *Géol*. *Lyon* **79**, 1–1184 (1980).

37. Granger, W. & Gregory, W. K. Further notes on the gigantic extinct rhinoceros *Baluchitherium* from the Oligocene of Mongolia. *Bull*. *Am*. *Mus*. *Nat*. *Hist*. **72**, 1–73 (1936).
38. Chiu, C.-S. A new genus of giant rhinoceros from Oligocene of Dzungaria, Sinkiang. *Vert*. *PalAsiat*. **11**, 182–191 (1973).
39. Forster-Cooper, C. On the skull and dentition of *Paraceratherium bugtiense,* a genus of aberrant rhinoceros from the lower Miocene deposits of Dera Bugti. *Phil*. *Trans*. *R*. *Soc*. *London* **212**, 369–394 (1924).
40. Pavlow, M. *Indricotherium transouralicum* n. sp. provenant du district de Tourgay. *Bull*. *Soc*. *Nat*. *Moscow* **31**, 95–116 (1922).
41. Gromova, V. Giant rhinoceroses. *Trav*. *Paleont*. *Inst*. *Acad*. *Sci*. *USSR* **71**, 1–164 (1959).
42. Li, Y.-X., Zhang, Y.-X., Li, J., Li, Z.-C. & Xie, K. New fossils of paraceratheres (Perissodactyla, Mammalia) from the early Oligocene of the Lanzhou Basin, Gansu Province, China. *Vert*. *PalAsiat*. **56**, 367–381 (2017).
43. Forster-Cooper, C. *Paraceratherium bugtiense*, a new genus of Rhinocerotidae from the Bugti Hills of Baluchistan. *Ann*. *Mag*. *Nat*. *Hist*. **8**, 711–716 (1911).
44. Chow, M.-C. & Chiu, C.-S. New genus of giant rhinoceros from Oligocene of Inner Mongolia. *Vert*. *PalAsiat*. **7**, 230–239 (1963).
45. Scott, W. B. & Osborn, H. F. The mammalia of the Uinta Formation. *Trans*. *Am*.

Philos. *Soc*. **16**, 461–572 (1890).

46. Wall, W. P. The genus *Amynodon* and its relationship to other members of the Amynodontidae (Perissodactyla, Rhinocerotoidea). *J. Paleont*. **56**, 434–443 (1982).

47. Borissiak, A. A. in *In Memory to the 50 Years of Scientific and Educational Activities of V. A. Obrutschev II*. 271–276 (Acad. Sci. USSR, Moscow, 1939).

48. Borsuk-Bialynicka, M. Studies on the Pleistocene rhinoceros *Coelodonta antiquitatis* (Blumenbach). *Palaeont*. *Polon.* **29**, 1–94 (1973).

49. Qi, G.-Q. Quaternary mammalian fossils from Salawusu River district, Nei Mongol. *Vert*. *PalAsiat*. **13**, 239–249 (1975).

50. Radinsky, L. B. A review of the rhinocerotoid family Hyracodontidae (Perissodactyla). *Bull*. *Am*. *Mus*. *Nat*. *Hist*. **136**, 1–45 (1967).

51. Lucas, S. G., Schoch, R. M. & Manning, E. The systematics of *Forstercooperia*, a middle to late Eocene hyracodontid (Perissodactyla, Rhinocerotoidea) from Asia and western North America. *J. Paleont*. **55**, 826–841 (1981).

52. Cope, E. D. The vertebrata of the Tertiary formations of the West. *Rep*. *U. S. Geol*. *Surv*. *Terr*. **3**, 657–691 (1884).

53. Scott, W. B. The mammalian fauna of the White River Oligocene, Part V. Perissodactyla. *Trans*. *Am*. *Philos*. *Soc*. **28**, 747–980 (1941).

54. Chow, M.-C. & Chiu, C.-S. An Eocene giant rhinoceros. *Vert*. *PalAsiat*. **8**, 264–268 (1964).

55. Wood, H. E. II. A primitive rhinoceros from the late Eocene of Mongolia. *Am*.

Mus. Novit. **2146**, 1–11 (1963).

56. Borissiak, A. A. *Indricotherium asiaticum* n. gen., n. sp. *Mem. Soc. Geol. France Paleont.* **35**, 1–128 (1923).

57. Forster-Cooperia, C. *Baluchitherium osborni* (? Syn. *Indricotherium turgaicum,* Borissiak). *Philos. Trans. R. Soc.* **212**, 35–66 (1923).

58. Xu, Y.-X. & Wang, J.-W. New materials of giant rhinoceros: Reports of paleontological expedition to Sinkiang (III): Permian and Triassic vertebrate fossils of Dzungaria Basin and Tertiary stratigraphy and mammalian fossils of Turfan Basin. *Mem. Inst. Vert. Paleont. Paleoanthr. Acad. Sin.* **13**, 132–140 (1978).

59. Wall, W. P. & Manning, E. *Rostriamynodon grangeri* n. gen., n. sp. of amynodontid (Perissodactyla, Rhinocerotoidea) with comments on the phylogenetic history of Eocene Amynodontidae. *J. Paleont.* **60**, 911–919 (1986).

60. Prothero, D. R. *The Evolution of North American Rhinoceroses* (Cambridge Univ. Press, Cambridge, 2005).

61. Hanson, C. B. in *The Evolution of Perissodactyls* (eds. Prothero, D. R. & Schoch, R. M.) 379–398 (Oxford Univ. Press, New York, 1989).

62. Russell, L. S. Tertiary mammals of Saskatchewan, VI: The Oligocene rhinoceroses. *Life Sci. Contrib. R. Ont. Mus.* **133**, 1–58 (1982).

63. Holbrook, L. T. & Lucas, S. G. A new genus of rhinocerotoid from the Eocene of Utah and the status of North American "*Forstercooperia*". *J. Vert. Paleont.* **17**, 384–396 (1997).

64. Chiu, C.-S. Giant rhinoceros from Loping, Yunnan, and discussion on the taxonomic characters of *Indricotherium grangeri*. *Vert. PalAsiat.* **6**, 57–71 (1962).

65. Cerdeño, E. Cladistic analysis of the Family Rhinocerotidae (Perissodactyla). *Am. Mus. Novit.* **3143**, 1–25 (1995).

66. Holbrook, L. T. The phylogeny and classification of tapiromorph perissodactyls (Mammalia). *Cladistics* **15**, 331–350 (1999).

67. Antoine, P.-O. Phylogénie et évolution des Elasmotheriina (Mammalia, Rhinocerotidae). *Mem. Mus. Natl. Hist. Nat.* **188**, 1–359 (2002).

68. Lu, X.-K. A juvenile skull of *Acerorhinus yuanmouensis* (Mammalia: Rhinocerotidae) from the late Miocene hominoid fauna of the Yuanmou Basin (Yunnan, China). *Geobios* **46**, 539–548 (2013).

69. Wang, H.-B., Bai, B., Meng, J. & Wang, Y.-Q. Earliest known unequivocal rhinocerotoid sheds new light on the origin of giant rhinos and phylogeny of early rhinocerotoids. *Sci. Rep.* **6**, 39607 (2016).

70. Groves, C. P. Phylogeny of the living species of rhinoceros. *J. Zool. Syst. Evol. Res.* **21**, 293–313 (1983).
