# Supplementary material for: An Oligocene giant rhino provides insights into Paraceratherium evolution
Source: Commun Biol. 2021 Jun 17;4:639. doi: 10.1038/s42003-021-02170-6 (PMC8211792; doi:10.1038/s42003-021-02170-6)
Supplement: Supplementary file 3 — Description of Supplementary Files [file 42003_2021_2170_MOESM3_ESM.pdf]

Description of Additional Supplementary Files

File name: Supplementary Data 1

Description: *List of samples used to code characters of taxa of this study.*

Abbreviations: AMNH, American Museum of Natural History, New York, USA; AMNH FM, Frick Collection of AMNH; CM, Carnegie Museum of Natural History, Pittsburgh, Pennsylvania, USA; DMNH, Denver Museum of Natural History (now Colorado Museum of Nature and Science), Denver, Colorado, USA; IVPP, Institute of Vertebrate Paleontology and Paleoanthropology, Chinese Academy of Sciences, Beijing, China; IVPP V, prefix to fossil vertebrates of IVPP ; IVPP O, prefix to living vertebrates of IVPP; ROM, Royal Ontario Museum, Toronto, Canada; MCZ, Museum of Comparative Zoology, Cambridge, Massachusetts, USA; NWUV, prefix to vertebrate specimens of Northwest University, Xi'an, China; SS, prefix to specimens of Sino-Soviet Palaeontological Expedition; IPN (ПИН), Borissiak Palaeontological Institute, Russian Academy of Sciences, Moscow, Russia; TMM, Texas Memorial Museum, Austin, Texas; TP, prefix to specimens of Turpan Museum, Turpan, China; UCMP, University of California Museum of Paleontology, Berkeley, California, USA; USNM, United States National Museum, Smithsonian Institution, Washington D.C., USA; UW, University of Washington Burke Museum, Seattle, Washington, USA; YPM, Yale Peabody Museum, Yale University, New Haven New Haven, Connecticut, USA; YPM-PU, Princeton University collection, now housed in the collections of YPM; ZAPUJ, prefix to specimens of Zoological Museum, Jagiellonian University, Kraków, Poland.

File name: Supplementary Data 2

Description: *Character list used in phylogenetic analysis.* The 155 characters used for our phylogenetic analysis are: 35 for cranium and mandible, 63 for teeth, and 57 for postcranial skeleton. Among them, 73 characters are new based mainly on the description[17], and other 82 characters are directly adopted or modified from previous phylogenetic analysis studies[65-69]. In this study, all characters are treated as non-additive.

File name: Supplementary Data 3

Description: *Dataset used in phylogenetic analysis.* The coding for 27 taxa, including all genera of giant rhinos and all species within the genus *Paraceratherium*. *Hyrachyus* was assigned as outgroup. Other taxa involved in the matrix cover the major groups once living in the Paleogene, Amynodontidae, Hyracodontidae, and Rhinocerotidae, the only clade surviving through the Neogene and Quaternary.
