# Supplementary material for: An Oligocene giant rhino provides insights into Paraceratherium evolution
Source: Commun Biol. 2021 Jun 17;4:639. doi: 10.1038/s42003-021-02170-6 (PMC8211792; doi:10.1038/s42003-021-02170-6)
Supplement: Supplementary file 7 — Reporting Summary [file 42003_2021_2170_MOESM7_ESM.pdf]

nature research

Corresponding author(s): Tao Deng

Last updated by author(s): Apr 26, 2021

Reporting Summary

Nature Research wishes to improve the reproducibility of the work that we publish. This form provides structure for consistency and transparency in reporting. For further information on Nature Research policies, see Authors & Referees and the Editorial Policy Checklist .

Statistics

For all statistical analyses, confirm that the following items are present in the figure legend, table legend, main text, or Methods section.

n/a	Confirmed	
☐	☒	The exact sample size (*n*) for each experimental group/condition, given as a discrete number and unit of measurement
☐	☒	A statement on whether measurements were taken from distinct samples or whether the same sample was measured repeatedly
☒	☐	The statistical test(s) used AND whether they are one- or two-sided<br>*Only common tests should be described solely by name; describe more complex techniques in the Methods section.*
☒	☐	A description of all covariates tested
☒	☐	A description of any assumptions or corrections, such as tests of normality and adjustment for multiple comparisons
☒	☐	A full description of the statistical parameters including central tendency (e.g. means) or other basic estimates (e.g. regression coefficient) AND variation (e.g. standard deviation) or associated estimates of uncertainty (e.g. confidence intervals)
☒	☐	For null hypothesis testing, the test statistic (e.g. *F*, *t*, *r*) with confidence intervals, effect sizes, degrees of freedom and *P* value noted<br>*Give P values as exact values whenever suitable.*
☒	☐	For Bayesian analysis, information on the choice of priors and Markov chain Monte Carlo settings
☒	☐	For hierarchical and complex designs, identification of the appropriate level for tests and full reporting of outcomes
☒	☐	Estimates of effect sizes (e.g. Cohen's *d*, Pearson's *r*), indicating how they were calculated

Our web collection on statistics for biologists contains articles on many of the points above.

Software and code

Policy information about availability of computer code

Data collection	Photographs of the fossils were taken using a Nikon D850 camera; 3D scanners for capturing the geometry and producing a three-dimension digital model of fossil were based on an Artec Spider; and the software using to collect data and establish matrix was Mesquite.
Data analysis	Adobe Photoshop CS 6 software was used to edit figures; Artec Studio software was used to reconstruct the 3D data; and TNT v1.5 software was used to analyse the data matrix for phylogeny.

For manuscripts utilizing custom algorithms or software that are central to the research but not yet described in published literature, software must be made available to editors/reviewers. We strongly encourage code deposition in a community repository (e.g. GitHub). See the Nature Research guidelines for submitting code & software for further information.

Data

Policy information about availability of data

All manuscripts must include a data availability statement. This statement should provide the following information, where applicable:

- Accession codes, unique identifiers, or web links for publicly available datasets
- A list of figures that have associated raw data
- A description of any restrictions on data availability

The authors declare that all data, including the photographs (Figures 1-7 and Supplementary Figures 1-3), measurements (Supplementary Tables 2-6), source data for paleogeographical analysis, and phylogenetic data matrix, which support the findings of this research, are provided in text or as supplementary information. The specimens (HMV 2006 and 2007) described in this study is archived and available on request from the Hezheng Paleozoological Museum in Hezheng County, Gansu Province, China.

Field-specific reporting

Please select the one below that is the best fit for your research. If you are not sure, read the appropriate sections before making your selection.

☐ Life sciences ☐ Behavioural & social sciences ☒ Ecological, evolutionary & environmental sciences

For a reference copy of the document with all sections, see nature.com/documents/nr-reporting-summary-flat.pdf

Ecological, evolutionary & environmental sciences study design

All studies must disclose on these points even when the disclosure is negative.

Study description	Our study describe a new giant rhino based on a complete skull and mandible with the associated atlas, and an axis and two thoracic vertebrae of another individual from the Late Oligocene (26.5 million years ago) deposits in northwestern China. Phylogenetic and palaeogeographical analyses were performed. Based on the dispersal of giant rhinos and the history of transgression-regression cycles of the Tethys Ocean in the Tarim Basin during Paleogene, we discussed the paleo-elevation of the Tibetan Plateau.
Research sample	Fossil and extant genera and species of the superfamily Rhinocerotoidea were used to compare with new fossils and to established the data matrix for cladistic analysis. Many localities situated south to or north to the Tibetan Plateau in Asia and a series of localities in the Tarim Basin were used to analyse paleogeography.
Sampling strategy	All samples from other species within the genus Paraceratherium are used for comparison. All genera of the giant rhino, other Paleogene rhinocerotoids (branching group), and additional 3 Neogene and Quaternary genera are used for the cladistic analysis to establish the phylogenetic relationship of the family Paraceratheriidae. Whenever possible, we selected taxa with well-preserved samples as well as distinct diagnosis to illustrate their shape outline, and included taxa once appeared in previous phylogenetic analyses to make a forceful comparison. We marked all localities with fossils of genus Paraceratherium in the paleogeographical analysis in order to display their dispersal history. In Supplementary Figure 3, we marked 21 classic localities in the Tarim Basin, from which typical formations with marine sediments and fossils have been discovered.
Data collection	The fossil specimens described in this study were collected by a joint team of the Institute of Vertebrate Paleontology and Paleoanthropology and the Hezheng Paleozoological Museum from the Late Oligocene deposits near Wangjiachuan Village in Dongxiang County, Gansu Province, China
Timing and spatial scale	The fossil specimens were discovered in May 2015, and prepared by three professional technicians from the Hezheng Paleozoological Museum under the supervision of the authors from December 2016 to February 2017. Since then, the specimens were studied by the authors.
Data exclusions	No data are excluded.
Reproducibility	The fossil specimens were housed in the Hezheng Paleozoological Museum, and are available on request; we provided detailed description of the methods and all the raw data, including character coding for phylogenetic analysis and measurements for comparison in the supplementary information, which can be used to reproduce all the results obtained in this study. The results reported in this study can be reproduced according to the methods clarified in the text.
Randomization	Randomization is not applicable to this study, because this research studied an Oligocene giant rhino and performed phylogenetic and paleogeographical analyses to study the phylogeny and dispersal of giant rhinos during the Eocene and Oligocene.
Blinding	Blinding is not applicable to this study.

Did the study involve field work? ☐ Yes ☒ No

Reporting for specific materials, systems and methods

We require information from authors about some types of materials, experimental systems and methods used in many studies. Here, indicate whether each material, system or method listed is relevant to your study. If you are not sure if a list item applies to your research, read the appropriate section before selecting a response.

Materials & experimental systems

n/a	Involved in the study	
☒	☐	Antibodies
☒	☐	Eukaryotic cell lines
☐	☒	Palaeontology
☒	☐	Animals and other organisms
☒	☐	Human research participants
☒	☐	Clinical data

Methods

n/a	Involved in the study	
☒	☐	ChIP-seq
☒	☐	Flow cytometry
☒	☐	MRI-based neuroimaging

Palaeontology

Specimen provenance	The fossil specimens described in this study were collected by a joint team of the Institute of Vertebrate Paleontology and Paleoanthropology and the Hezheng Paleozoological Museum from the Late Oligocene deposits near Wangjiachuan Village in Dongxiang County, Gansu Province, China in May 2015, and prepared by three professional technicians from the Hezheng Paleozoological Museum under the supervision of the authors from December 2016 to February 2017.
Specimen deposition	The specimens are now housed in the Hezheng Palaeozoological Museum under the catalog numbers HMV 2006 and 2007, and available on request.
Dating methods	No new dates are provided.

☒ Tick this box to confirm that the raw and calibrated dates are available in the paper or in Supplementary Information.
